# Tailored chemokine receptor modification improves homing of adoptive therapy T cells in a spontaneous tumor model

**DOI:** 10.18632/oncotarget.9280

**Published:** 2016-05-10

**Authors:** Stefano Garetto, Claudia Sardi, Elisa Martini, Giuliana Roselli, Diego Morone, Roberta Angioni, Beatrice Claudia Cianciotti, Anna Elisa Trovato, Davide Giuseppe Franchina, Giovanni Francesco Castino, Debora Vignali, Marco Erreni, Federica Marchesi, Cristiano Rumio, Marinos Kallikourdis

**Affiliations:** ^1^ Adaptive Immunity Laboratory, Humanitas Clinical and Research Center, Rozzano (Milano), Italy; ^2^ Humanitas Clinical and Research Center, Rozzano (Milano), Italy; ^3^ Laboratory of Cellular Immunology, Humanitas Clinical and Research Center, Rozzano (Milano), Italy; ^4^ Dipartimento di Biotecnologie Mediche e Medicina Traslazionale, Università degli Studi di Milano, Milan, Italy; ^5^ Dipartimento di Scienze Farmacologiche e Biomolecolari, Università degli Studi di Milano, Milan, Italy; ^6^ Humanitas University, Rozzano (Milano), Italy

**Keywords:** tumor, Adoptive Cell Therapy, T cells, chemokines, fibrosis

## Abstract

In recent years, tumor Adoptive Cell Therapy (ACT), using administration of *ex vivo*-enhanced T cells from the cancer patient, has become a promising therapeutic strategy. However, efficient homing of the anti-tumoral T cells to the tumor or metastatic site still remains a substantial hurdle. Yet the tumor site itself attracts both tumor-promoting and anti-tumoral immune cell populations through the secretion of chemokines. We attempted to identify these chemokines in a model of spontaneous metastasis, in order to “hijack” their function by expressing matching chemokine receptors on the cytotoxic T cells used in ACT, thus allowing us to enhance the recruitment of these therapeutic cells. Here we show that this enabled the modified T cells to preferentially home into spontaneous lymph node metastases in the TRAMP model, as well as in an inducible tumor model, E.G7-OVA. Due to the improved homing, the modified CD8^+^ T cells displayed an enhanced *in vivo* protective effect, as seen by a significant delay in E.G7-OVA tumor growth. These results offer a proof of principle for the tailored application of chemokine receptor modification as a means of improving T cell homing to the target tumor, thus enhancing ACT efficacy. Surprisingly, we also uncover that the formation of the peri-tumoral fibrotic capsule, which has been shown to impede T cell access to tumor, is partially dependent on host T cell presence. This finding, which would be impossible to observe in immunodeficient model studies, highlights possible conflicting roles that T cells may play in a therapeutic context.

## INTRODUCTION

The current standard therapeutic strategy for primary cancer patients is surgical ablation and/or chemotherapy. For tumors, or -more often- their metastases, that are refractive to these treatments, Adoptive Cell Therapy (ACT) using administration of cytotoxic CD8^+^ T cells, engineered to improve their specificity for tumor antigens, is producing increasingly promising results [1]. Yet, even at the experimental level, ACT is far from perfect. Extensive research efforts are gradually shedding light into different mechanisms of local immunosuppression at the tumor site. These mechanisms, which range from immunosuppressive regulatory T cells (Treg) and Myeloid-Derived Suppressor cells to metabolic changes that inhibit cytotoxic T cell activity [2, 3], will have to be overcome in order for the transferred CD8^+^ T cells to efficiently attack the tumor. Indeed, a combination of improved identification of tumor antigens and strategies designed to combat immunosuppression is yielding encouraging results [1]. Yet there still remains a substantial hurdle in ensuring that the transferred anti-tumoral T cells physically reach and penetrate the tumor or metastatic site. This relatively under-studied issue is likely to become a bottleneck in any cell-based therapeutic strategy [4].

Cells with pro-tumoral activity are recruited to the tumor via the action of chemokines and their receptors. In ACT, transferred CD8^+^ T cells may not necessarily express the chemokine receptors required to reach their target, or may do so at levels insufficient to respond to biochemically-modified chemokines released by the tumor site [5]. Within lymphoid structures, T cells have been shown to move along a dispersed reticular fiber network, thus gaining mobility within the tissue. Conversely, a recent pioneering imaging study demonstrated that human lung tumors are surrounded by a barrier of dense fibrous structures, parallel to the circumference of the tumor mass, which inhibit T cell entry [6, 7]. Indeed, a fibrotic barrier is thought to restrict access to therapeutic agents in lung, pancreatic and breast tumors [2, 8, 9]. Whilst such a capsule could initially restrain the tumor, dense collagen structures in breast cancer favor tumor progression: the extracellular matrix induces activating signals in tumor cells, leading to more aggressive and metastasis-prone tumors. The spatial organization of the collagen fibers gradually changes from dense to parallel barrier-like (which can shelter the tumor from immune attack as well as promote tumor progression to more aggressive states), and to a final more metastasis-prone radial arrangement [9]. Thus, at least in some tumors, a fibrotic capsule may inhibit immunosurveillance, giving the tumor time to become more aggressive. Highly metastatic tumor cell lines from various tumors have been shown to correlate with enhanced ability to “break through” collagen membranes mediated by collagenase enzymes [10], suggesting a mechanism for tumor escape in late stages. In an experimental mouse spontaneous pancreatic ductal adenocarcinoma, genetic depletion of the fibrous stroma led to a relative enrichment of infiltrating Treg, resulting in tumor growth [2]. In a clinical context of ACT, the excluded T cells would be more likely to be transferred cytotoxic CD8^+^ T cells rather than Treg. Salmon and co-workers indeed have demonstrated that *ex vivo* collagenase treatment enables excluded cytotoxic T cells to penetrate the tumor mass. Further, they have shown that the intact fibrotic barrier can be overcome by cytotoxic T cells when the xenotransplanted human tumor used as a target was made to overexpress the chemokine CCL5 [6]. These seminal findings have brought insight into the processes inhibiting efficient migration of anti-tumor T cells to the target site in ACT; yet they do not directly translate to therapy proposals. For this reason, clinically-relevant proof-of-principle solutions are still needed.

A strategy that has potential for translation to the clinic involves ectopically expressing a chemokine receptor on the T cells that can force their recruitment to the target site. As T cells are virally transduced in most ACT protocols in order to modify their specificity towards tumor-associated antigens [1], addition of a chemokine receptor-expressing vector can be achieved with minimal modifications to ACT protocols. In this context, chemokine receptors have been shown, by us and others, to be able to re-direct T cell migration in physiological *in vivo* conditions [11], *in vitro* towards chemokines detected in tumors [12], as well as *in vivo* towards implanted tumors [13–15]. To further the translational relevance of this strategy, it would be important, as a proof of principle, to tailor the approach to spontaneous tumors. For this, here we utilized the transgenic adenocarcinoma of mouse prostate (TRAMP), a mouse model of prostate cancer, one of the tumors with highest associated mortality [16]. Male TRAMP mice closely mirror the pathology of human prostate cancer and, importantly, spontaneously form lymph node and lung metastases [17]. We analyzed the chemokine expression pattern of the lymph node metastases in TRAMP mice. We identified the chemokine most robustly expressed in the spontaneous metastatic lymph nodes, cloned a vector encoding its matching chemokine receptor and utilized it to transduce CD8^+^ T cells, along with constructs encoding for tumor-specific T cell receptors. This enabled the modified T cells to preferentially home into metastatic lymph nodes, as demonstrated by flow cytometry and 2-photon microscopy. We show that the modified CD8^+^ T cells maintain intact their *in vitro* killing capacity, whilst, due to the improved homing, they display an improvement in *in vivo* anti-tumor activity, as seen by a delay in tumor growth. Thus chemokine receptor-modified T cells can enable CD8^+^ T cells in ACT to gain enhanced access to the tumor. Surprisingly, by examining the levels of tumor-associated fibrosis in mice lacking T cells, we uncover that the peri-tumoral fibrotic capsule, which can impede T cell access [6] and is thus part of the obstacles to therapy, is partially dependent on host T cell presence for its formation. This finding, which would not be possible to observe in immunodeficient xenotransplantation models, highlights the intriguing possibility that T cells in a therapeutic context may play conflicting roles.

## RESULTS

### CCL2 expression is robustly upregulated in sites of spontaneous lymph node metastasis in TRAMP

**Figure 1:**
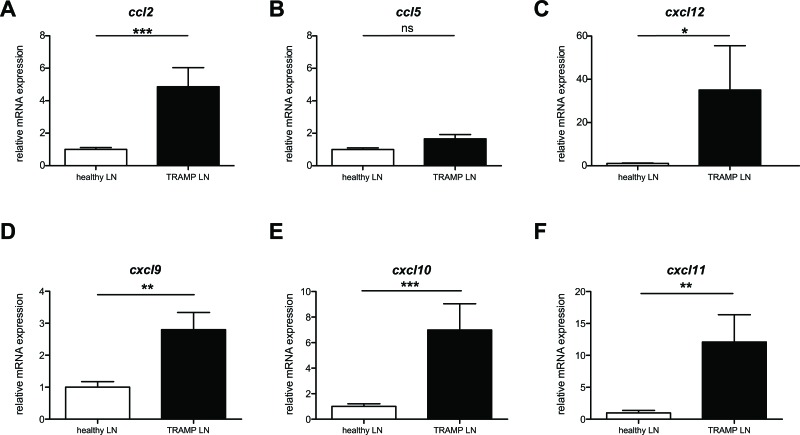
*Ccl2* mRNA is differentially upregulated in TRAMP metastatic lymph nodes **A**: *Ccl2* mRNA relative expression in TRAMP SV40^+^ or healthy lymph nodes; Mann Whitney test: (***) P<0.001. **B**: *Ccl5* mRNA relative expression in TRAMP SV40^+^ or healthy lymph nodes; Mann Whitney test: (ns) P>0.05. **C**: *Cxcl12* mRNA relative expression in TRAMP SV40^+^ or healthy lymph nodes; Mann Whitney test: (*) P< 0.05. **D**: *Cxcl9* mRNA relative expression in TRAMP SV40^+^ or healthy lymph nodes; Mann Whitney test: (**) P< 0.01. **E**: *Cxcl10* mRNA relative expression in TRAMP SV40^+^ or healthy lymph nodes; Mann Whitney test: (***) P< 0.001. **F**: *Cxcl11* mRNA relative expression in TRAMP SV40^+^ or healthy lymph nodes; Mann Whitney test: (**) P< 0.01.

Tumors secrete a number of different chemokines, which can mediate both the metastasis of the tumor itself as well as the recruitment and/or retention of cells with pro- or anti-tumoral function [18]. We hypothesized that by identifying the existing chemokine gradients in a spontaneous tumor metastasis, we would be able to “hijack” the gradient in order to enhance the migration of adoptively transferred anti-tumoral CD8^+^ cytotoxic T cells. The primary tumor in prostate cancer is usually surgically removed, thus rendering more clinically relevant an ACT treatment for metastasis rather than primary tumor. TRAMP mice spontaneously form lymph node and lung metastases [17]. We performed pilot experiments to identify the timing with which the spontaneous lymph node metastases in TRAMP occur, as the literature is equivocal on this point. We identified that at 26 weeks of age a high (but variable) percentage of TRAMP male mice developed metastasis in the inguinal and para-aortic lymph nodes. To identify the presence of metastasis, we analyzed all explanted lymph nodes by real-time qPCR and immunohistochemistry (IHC) (Supplemental Figure 1) for the expression of the SV40 large T antigen (SV40 TAg), which is part of the transgene driving oncogenesis [17]. SV40 TAg expression guarantees tumor presence, though its absence may indicate either lack of tumor or tumor that has lost expression of the antigen. Thus in all our assays we only considered SV40 TAg^+^ lymph nodes as metastatic and used age-matched healthy C57BL/6 lymph nodes rather than TRAMP SV40 TAg^−^ lymph nodes as healthy controls. Human and murine prostate tumors have been previously reported to express CCL2, CCL5 and CXCL12 [18–24]. We thus examined, by real-time qPCR, the expression of these chemokines in metastatic lymph nodes. We also assessed the expression of other chemokines, such CXCR3 ligands, that have been associated with inflammation and primary cancers [25]. CCL2 mRNA displayed extremely statistically significant upregulation in metastatic versus healthy lymph nodes (P<0.001, Mann Whitney test) (Figure 1A). CXCR3 ligands also displayed potentially exploitable differences in expression, especially CXCL10 (Figure 1D-F). Results for the primary tumor mass in the prostate reflected the same trend, albeit with lower statistical significance (Supplemental Figure 2A). On the basis of these results, either CCR2 or CXCR3, as cognate receptors for CCL2 and CXCL10 respectively, could be equally used in chemokine receptor-modified ACT. Given the extensive reports on CCL2 expression in prostate cancer [22, 24, 26], we chose to utilize CCR2.

### ACT with concurrent transduction of the chemokine receptor for CCL2 boosts homing of CD8^+^ T cells to the site of spontaneous metastasis in TRAMP

We thus next set out to perform ACT using CD8^+^ T cells from healthy C57BL/6 donors transduced with a T cell receptor construct conferring specificity for the antigen SV40 TAg, which is expressed in TRAMP [27]. Concurrently, we co-transduced a retroviral vector expressing CCR2 or a GFP control vector. The CCR2-expressing vector was capable of enhancing *in vitro* migration towards CCL2 (Supplemental Figure 2B) compared to control-transduced cells; this is unsurprising, as CCR2 is known to be expressed at very low levels in T cells [5].

**Figure 2:**
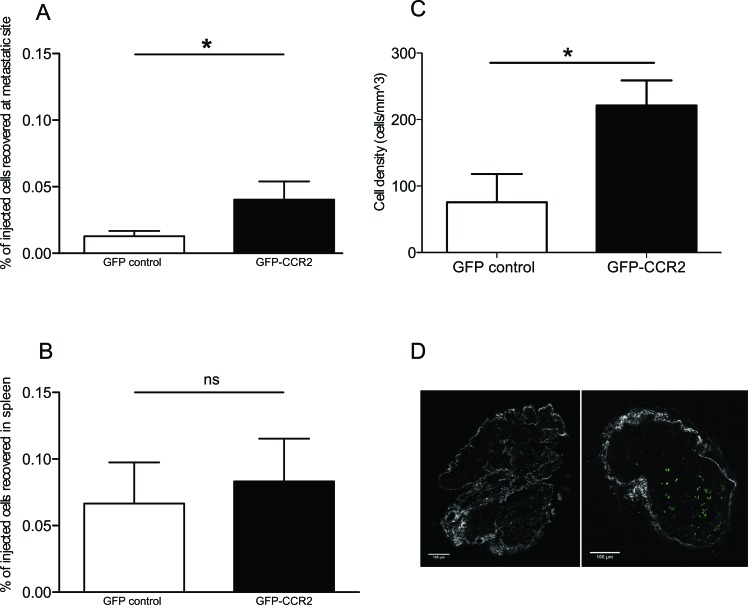
CCR2 transduction significantly improves homing to the metastatic lymph nodes **A**: percentage of GFP-CCR2 (n=13) or GFP-control (n=9) injected cells recovered from TRAMP SV40 TAg^+^ lymph nodes, 24 hours after adoptive transfer, analyzed by FACS; Mann Whitney test: (*) P<0.05. **B**: percentage of GFP-CCR2 (n=9) or GFP-control (n=10) injected cells, recovered from TRAMP spleens, 24 hours after adoptive transfer, analyzed by FACS; Mann Whitney test: (ns) P>0.05. **C**: density of GFP-CCR2 (n=4) versus GFP-control (n=6) in TRAMP SV40 TAg^+^ lymph nodes (cells per mm^3^), 24 hours after adoptive transfer, analyzed by 2-photon microscopy; unpaired t test: (*) P<0.05 (top). **D**: Representative images of SV40 TAg^+^ lymph nodes acquired by 2-photon microscopy. Lymph node from a recipient of GFP-control CD8^+^ T cells on the left; from a recipient of GFP-CCR2 CD8^+^ T cells on the right. Green signal indicates GFP^+^ cells; collagen stain by SHG shown in white.

The transduced cells were thus administered to 26 week-old TRAMP male ACT recipients. To examine whether CCR2 transduction improved T cell homing to TRAMP metastatic lymph nodes, we assessed by flow cytometry the presence of GFP^+^ cells in single cell suspensions of recipient lymph nodes harvested 24 hours after T cell transfer. Cells from each analyzed lymph node were examined in parallel by qPCR for SV40 TAg expression, as above, to confirm that they were indeed metastatic. The concurrent transduction with CCR2 significantly improved homing to metastatic lymph nodes (P<0.05, Mann Whitney test) (Figure 2A), whilst this was not so in off-target tissues such as the spleen (Figure 2B). A number of recipient lymph nodes were analyzed *ex vivo* by 2-photon microscopy, enabling the visualization of GFP^+^ cells. Comparison of the number of GFP^+^ cells in CCR2-transduced versus control-transduced T cell recipients confirmed that indeed CCR2 transduction significantly improves homing to the SV40 TAg^+^ metastatic lymph nodes (P<0.05, unpaired t test) (Figure 2C, 2D). Double staining of lymph node sections for tumor cells and T cells (using anti-SV40 TAg and anti-GFP antibodies) identified that the CCR2-transduced T cells were in contact with tumor cells (Supplemental Figure 3).

### Chemokine receptor transduction of CD8^+^ T cells does not affect their cytotoxic potential or state of activation

**Figure 3:**
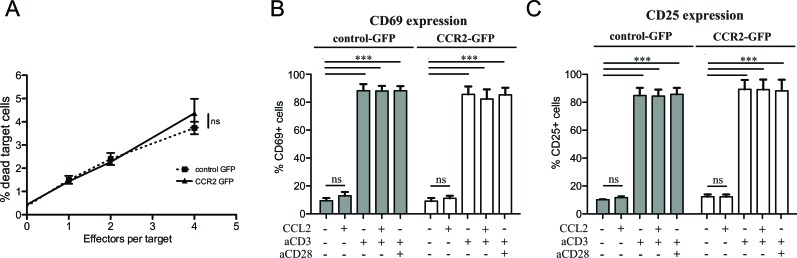
Chemokine receptor transduction does not affect killing capacity or activation state of CD8^+^ T cells **A**: Lack of effect of chemokine receptor transduction in *in vitro* T cell-mediated cytotoxicity. Target (5×10^4^ E.G7-OVA) cells were co-incubated with increasing concentrations of OVA-specific TCR/GFP-control or OVA-specific TCR/GFP-CCR2 transduced CD8^+^ T cells as effector cells. The target cells were labeled with Orange CMTMR Dye and cell death was assessed by 7-AAD. The percentage of dead target cells was evaluated by flow cytometry; 2-way repeated measures ANOVA: (ns) (P>0.05). **B**: Lack of costimulatory effects of chemokine ligand on chemokine receptor-transduced CD8^+^ T cells. CCL2 costimulation assays were performed with GFP-CCR2 or GFP-control-transduced CD8^+^ T cells. Activation markers CD25 and CD69 were measured by flow cytometry 18h after stimulation. 2-way ANOVA: (***) P<0.001; (ns) P>0.05.

In order to assess whether the improvement in homing is reflected in *in vivo* anti-tumor activity, we decided to monitor tumor growth *in vivo* during ACT with CCR2- or control-transduced CD8^+^ T cells. The metastasis in the lymph nodes of TRAMP mice does not form well-defined solid structures, which would be necessary to enable quantification of tumor size *in vivo*. For this reason, we opted to utilize an injectable tumor cell line. Whilst TRAMP-derived cell lines do exist, they have lost the expression of the SV40 large T antigen, thus rendering a “standard” ACT using T cells transduced with a tumor-specific TCR impossible. We thus chose to inject C57BL/6 mice with the tumor cell line E.G7-OVA, a lymphoma cell line expressing the antigen ovalbumin as well as similar levels of CCL2 compared with TRAMP metastatic lymph nodes (Supplemental Figure 4). To rule out any possible negative functional side-effects of chemokine receptor transduction on the CD8^+^ T cells, we performed *in vitro* T cell-mediated cytotoxicity assays, using E.G7-OVA cells as targets (Figure 3A). The CD8^+^ T cells were transduced with a TCR specific for ovalbumin [28] as well as constructs for CCR2 or a control vector. The results confirmed that CCR2 transduction does not negatively affect CD8^+^ T cell cytotoxic function.

A number of chemokines have been shown to be able to provide costimulation signals to T cells, leading to enhanced T cell activation [29]. This has also been shown to occur with CCL2 [30, 31]. Thus we could not exclude the possibility that transduction of CCR2 in CD8^+^ T cells could enable them to receive costimulatory signals from CCL2, in addition to improving their homing towards CCL2. To test this, we set up *in vitro* costimulation assays, using T cells that had been transduced with CCR2 or control vectors. The assays were performed 48h after transduction, so as to match the timing of injection for ACT. We found that CCL2 did not have any costimulatory effect on the T cells, as judged by expression of activation markers CD69 and CD25 (Figure 3B, 3C). However, this does not preclude that CCL2 *per se* cannot mediate costimulation. Rather, it is likely that the activation and costimulation received by the T cells in order to transduce them in the first place (see Materials and Methods), renders any further costimulation unnecessary or impossible, as indeed is the case for pre-activated T cells [32]. Indeed, in our assays, costimulation of the transduced cells was not evident even with the administration of “classical” anti-CD28 costimulatory signals (Figure 3B, 3C). Thus it is unlikely that CCL2 may be costimulating the transferred T cells in our experimental ACT setup.

### CD8^+^ cells transduced for CCR2 infiltrate the tumor mass and significantly delay tumor growth *in vivo*

**Figure 4:**
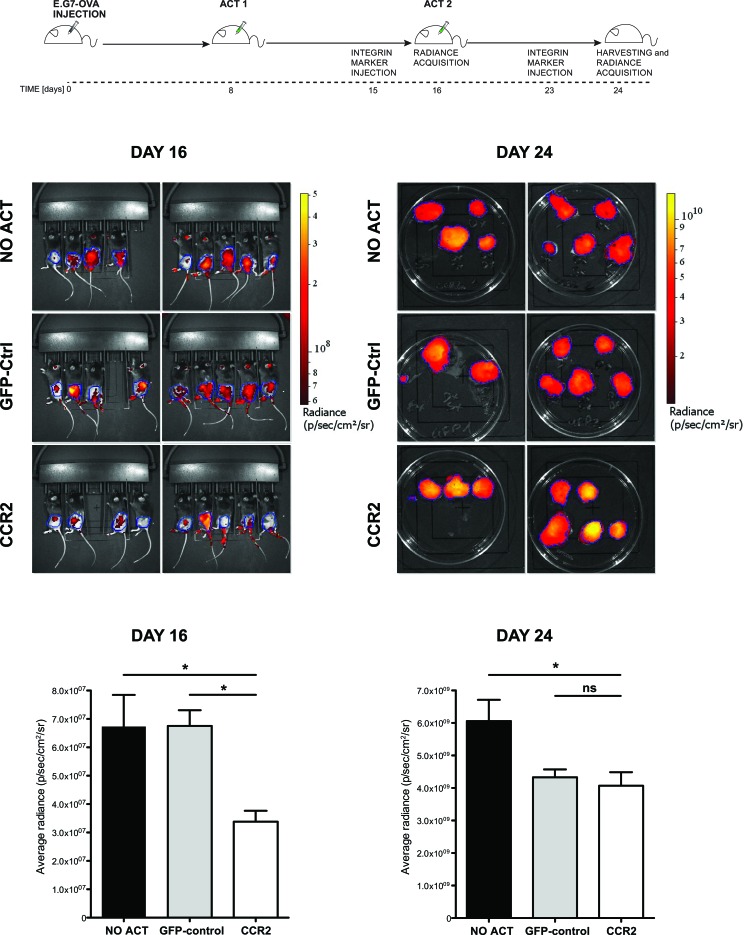
Chemokine receptor-modified CD8^+^ T cells lead to significant delay in tumor growth Top: experimental design scheme. Images and plots: (DAY 16) *in vivo* Integrisense radiance emission detection of implanted E.G7-OVA tumor, 8 days after the first adoptive transfer of CCR2 (n=8) or GFP-control (n=8) transduced cells or no transfer (n=9); 1-way ANOVA and Tukey's multiple comparison test: (*) P<0.05. (DAY 24) Integrisense radiance emission of harvested E.G7-OVA tumor, 8 days after the second adoptive transfer of CCR2 (n=8) or GFP-control (n=7) transduced cells or no transfer (n=9); 1-way ANOVA and Tukey's multiple comparison test: (*) P<0.05. Data are displayed as mean + SEM of radiance for each group of animals at a given time point.

We next proceeded to perform ACT on mice that had received E.G7-OVA tumors subcutaneously. In order to remain as close as possible to clinically-relevant limitations, we utilized a mild ACT regimen, with no concurrent administration of adjuvants, using T cells transduced with CCR2 or control vectors as well as a vector expressing ovalbumin-specific TCR. The latter choice, despite its lower efficiency in rendering tumor antigen-specificity compared to transgenic antigen-specific T cells, was preferred due to its vicinity to clinically applicable solutions. Mice were divided into three groups, each group receiving CCR2- or control-transduced CD8^+^ T cells or no ACT. A first T cell administration was performed 8 days after tumor injection, as shown in the schematic outline (Figure 4, schematic diagram). Tumors were monitored by *in vivo* fluorescence detection of an integrin marker. 8 days after the first ACT (Figure 4 – day 16), we observed a significant reduction (close to 50%) in tumor mass in the group treated with T cells transduced with OVA-specific TCR/CCR2 compared to tumors in mice that received no ACT or mice that received T cells transduced only with the TCR and GFP-control vectors. Thus the chemokine receptor-modified CD8^+^ T cells exhibited an improved control over tumor growth. Administration of further T cells with a second ACT eliminated the difference between CCR2- and control-transduced T cells by day 24, though CCR2-transduced T cells still resulted in a significant –albeit poor- restraining of tumor growth (Figure 4 – day 24). We speculate that the aggressive tumor growth by day 24, which is not unexpected given the “weak” ACT regimen followed, may account for the loss of significant difference between the two treatments. Nonetheless, these results demonstrate that tailored modification of the chemokine receptor in CD8^+^ T cells used in ACT can have a significant effect in slowing down tumor growth.

**Figure 5:**
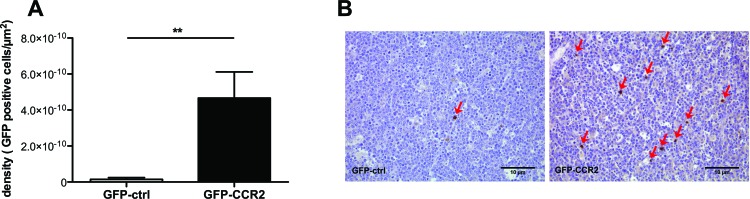
Enhancement in ACT efficacy is associated with improved homing of chemokine receptor-modified CD8^+^ T cells **A**: Density of injected cells in E.G7-OVA implanted tumors (cells per μm^2^), at the end of therapy; Mann Whitney test, recipients of GFP-CCR2 (n=8) versus GFP-control (n=7) transduced T cells: (**) P<0.01 **B**: Representative images of immunohistochemical analysis: Brown staining/red arrows: GFP^+^ cells (GFP-ctrl^+^ or GFP-CCR2^+^ CD8^+^ T cells).

As CCR2-transduced CD8^+^ T cells do not differ in *in vitro* cytotoxic capacity from control-transduced cells, any beneficial effect on tumor growth was likely to be due to improved homing. Indeed, when we evaluated by immunohistochemistry the presence of GFP^+^ cells in the implanted E.G7-OVA tumors, we observed a significantly greater number of cells in the recipients of CCR2-transduced compared to control-transduced CD8^+^ T cells (Figure 5A, 5B). This result confirms, in a different tumor model, that tailored chemokine receptor modification improves T cell homing, matching our findings in TRAMP spontaneous metastatic lymph nodes.

### TRAMP metastatic sites are characterized by increased fibrosis

The pro-tumoral barrier function of the fibrous formations of collagen is usually associated with pancreatic, lung and breast tumors [2, 8, 9]. The metastatic lymph nodes in the TRAMP model would clearly not pose any such barrier to T cell entry, as the lymph node is physiologically accessible to lymphocytes. Nonetheless, we enquired whether the increased collagen deposition associated with the fibrosis-prone tumors could also be observed in these spontaneous prostate cancer metastases. Utilizing second harmonic generation in 2-photon microscopy, which enables visualization of collagen *in situ*, we examined collagen deposition in SV40 TAg^+^ metastatic or healthy lymph nodes. The metastatic lymph nodes had significantly higher levels of collagen (Supplemental Figure 5), suggesting that cancer-associated fibrosis could be associated with tumors not considered “fibrosis-prone”.

### The presence of T cells reduces tumor size but favors peri-tumoral fibrosis formation

**Figure 6:**
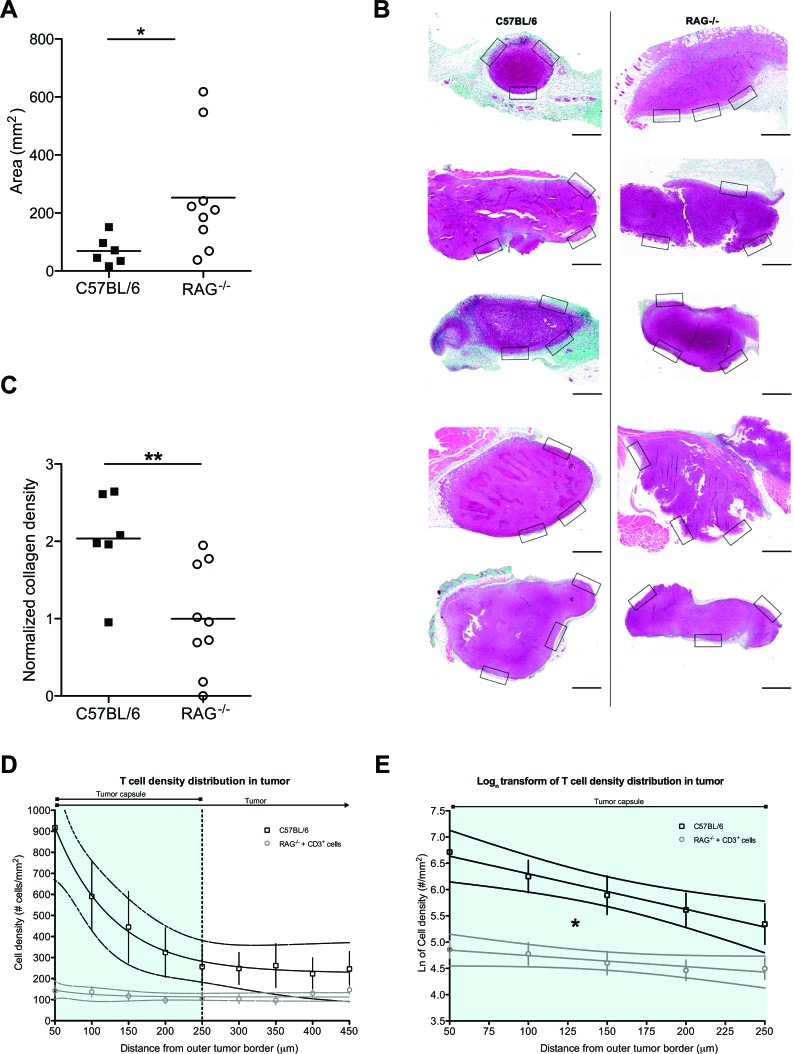
Host T cell presence inhibits tumor growth but simultaneously favors fibrosis formation **A**: PANC02 tumor size, shown as tumor area after 3 weeks of *in* vivo growth, in the presence (C57BL/6J recipients) or absence (RAG2^−/−^ recipients) of host T cells; Mann Whitney test: (*) P<0.05. Each dot represents one animal. **B**: Representative images of Masson's trichrome staining of harvested PANC02 tumor sections. Collagen stain visible in green/cyan color. Boxes show the areas from which regions of interest were obtained to calculate mean collagen density for every sample. Scale bars represent 50 μm. **C**: collagen density in the presence (C57BL/6J recipients) or absence (RAG2^−/−^ recipients) of host T cells; Mann Whitney test (**) P<0.01. Each dot represents one animal. **D**: T cell density distribution across PANC02 tumors grown for 3 weeks in C57BL/6 recipients that are T cell-sufficient (n=5) or in RAG2^−/−^ recipients in which CD3^+^ cells were injected 3 days prior to analysis (n=8). Y-axis values represent the mean T cell density ± SEM in concentric regions starting from the outer tumor capsule border and moving towards the center of the tumor (examples of the regions shown in Supplemental Figure 7). X-axis values show the distance of the region from the outer tumor capsule border. The vertical dotted line marks the estimated average inner edge of the tumor capsule, derived from 2PM SHG imaging. 95% confidence intervals are also shown. The slope of the RAG2^−/−^ recipients is not different from a flat line (P>0.05, linear regression). **E**: Natural logarithmic transform of the T cell distributions, for the region covering the average capsule width (0-250 μm from the outer tumor border). Data points shown as mean ± SEM; 95% confidence intervals are also shown. The two slopes are significantly different (*: P<0.05, linear regression).

A putatively more ubiquitous presence of fibrosis associated with tumors led us to form a hypothesis as to why such structures may form within diffuse tumors or around solid tumors. Tumors are thought to behave as never-healing wounds [33]. Intriguingly, T cells have been suggested to play an important role in fibrosis formation during wound healing [34], whilst Th2-polarized cells correlate with tumor-promoting inflammation [35]. We thus hypothesized that (host) T cells could be contributing to the formation of tumor-associated fibrosis. To test this, we injected cells from a pancreatic tumor cell line, PANC02, into T and B cell-deficient RAG2^−/−^ mice or wild-type C57BL/6 controls. After 3 weeks of tumor growth, we examined the size of the explanted tumors as well as the formation of a fibrotic capsule around the solid tumors. Unsurprisingly, given the well-characterized role of T cells in immunosurveillance [36], RAG2^−/−^ recipients developed significantly larger tumors than the wild-type recipients (Figure 6A). Surprisingly, however, the tumors in RAG2^−/−^ mice had significantly less dense peri-tumoral collagen than wild-type mouse tumors, as assessed by Masson's trichrome staining (Figure 6B, 6C), as well as by second harmonic generation in 2-photon microscopy (Supplemental Figure 6). This suggests that T cell presence contributes to the formation of the tumor-associated fibrotic capsule.

As mentioned above, the peri-tumoral fibrotic capsule has been shown to inhibit T cell infiltration into tumors [2, 6]. We thus asked whether the reduction in collagen density that we observed in 3-week tumors from T cell-deficient RAG2^−/−^ mice would lead to greater ease of infiltration by T cells. To assess this, we had to compare T cell infiltration in tumors grown in the presence or in the absence of T cells. We thus first analyzed sections of tumors extracted after 3 weeks of growth in control T cell-sufficient C57BL/6 tumor recipients. We examined the distribution of T cells across the tumor, identifying the presence T cells via immunohistochemistry for CD3 (Figure 6D, upper line and Supplemental Figure 7). On the other hand, in the absence of T cells (i.e. in RAG2^−/−^ tumor recipients), the host has no T cells that may infiltrate the tumor. Thus to examine T cell infiltration in tumors grown in the absence of T cells, we transferred 3×10^6^ CD3^+^ T cells into a cohort of PANC02 tumor-bearing RAG2^−/−^ mice, 3 days before harvesting their 3-week-old PANC02 tumors. To facilitate the identification of the transferred T cells, we stained them with Cell Tracker Orange CMTMR dye prior to injection. Using 2-photon microscopy on the extracted tumor sections, we identified transferred T cells via their fluorescence and peri-tumoral capsules via second harmonic generation (Supplemental Figure 7). Staining with CMTMR introduces an uncontrolled variable, which could affect the total number of T cells measured. Yet, we were interested in how T cells were distributed across the tumor (the gradient of the distribution of T cells), a parameter that is both independent of the absolute number of total T cells present (within a physiological range of cell concentrations) as well as independent of the method of detection of the T cells in each group. Indeed, the total number of transferred T cells present in the RAG2^−/−^ recipients would be expected to be lower than the total number of T cells present in control T cell-sufficient hosts. Reflecting this, the total number of T cells detected in RAG2^−/−^ recipient tumors was lower than in C57BL/6 tumors (Figure 6D, upper versus lower lines). Yet, when we applied an image analysis algorithm on both groups (see Methods) to assess the way in which T cell density distribution changed along concentric regions starting from the outer tumor border and moving towards the center of the tumor, C57BL/6 tumors produced a cell density distribution that fit an exponential decay regression model (Figure 6D, upper line). This shows that a decreasing number of T cells are found deeper within the tumor, suggesting that the tumor capsule in C57BL/6 (the region marked “tumor capsule”, corresponding to the outermost 250 μm of the tumors in Figure 6D) impedes the infiltration of T cells. On the other hand, T cell density distribution across the tumor in RAG2^−/−^ mice fit a linear model, with a slope that did not significantly differ from a flat line (P>0.05, linear regression) (Figure 6D, lower line). This flat distribution suggests that in tumors grown in T cell-less recipients, possibly due to the lower density collagen of the tumor capsule, introduced T cells can move into the tumor without the impediment observed in control tumors. Finally, in order to compare the two rates of decay, we performed a logarithmic transform of the distribution data and applied a linear regression model, restricted to the region of the tumor capsule (Figure 6E). The two slopes were significantly different (P<0.05, linear regression). This signifies that the “flat” distribution of infiltrating T cells in RAG2^−/−^ tumors differs significantly from that of control mice. Thus T cell infiltration across tumor capsules in T cell-sufficient mice appears to be inhibited in a manner that is absent from T cell-deficient mice. Thus, taken together, the above suggest that (host) T cells contribute to capsule formation, which may obstruct T cell infiltration of the tumor.

**Figure 7:**
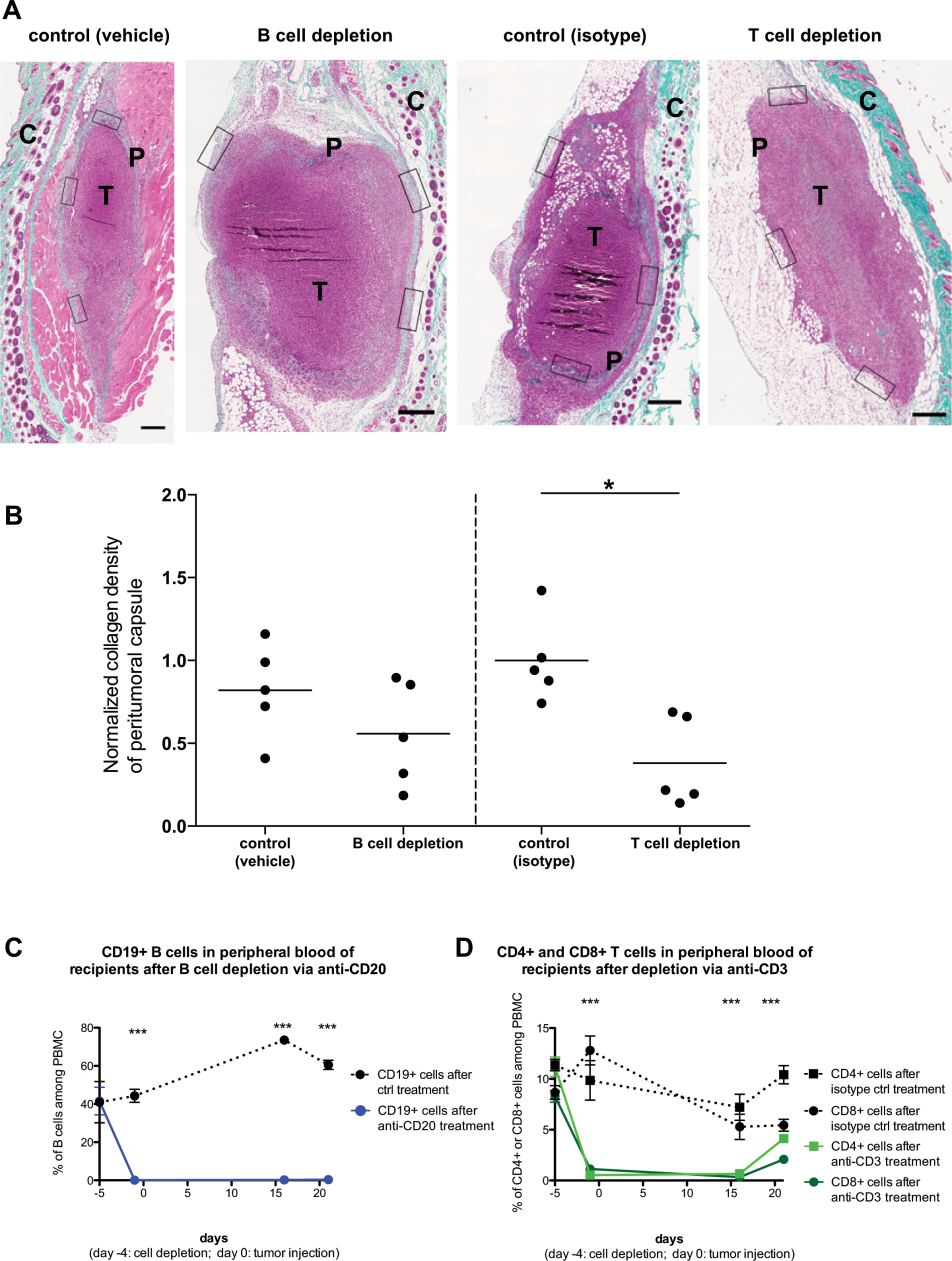
Host T cell but not B cell depletion significantly affects peritumoral fibrosis formation PANC02 tumors were implanted into C57BL/6J recipients, after depletion of B cells (via anti-CD20 administration) or T cells (via anti-CD3 administration). **A**: Representative images of Masson's trichrome staining of harvested PANC02 tumor sections, after 3 weeks of *in* vivo growth. Collagen stain visible in green/cyan color. Boxes show the areas from which regions of interest were obtained to calculate mean collagen density for every sample. Scale bars represent 50μm. “T” marks the tumor mass, “P” marks the peritumoral capsule, “C” marks the irrelevant fibrotic area associated with cutaneous structures (skin). **B**: collagen density in B cell-depleted recipients (and controls) or T cell-depleted recipients (and controls); 1-way ANOVA and Tukey's multiple comparison test: (*) P<0.05. Each dot represents one animal (n=5 per group). **C**: The efficiency of B cell depletion via anti-CD20 treatment, as measured by FACS analysis of blood samples for B cell marker CD19. **D**: The efficiency of T cell depletion via anti-CD3 treatment, as measured by FACS analysis of blood samples for T cell markers CD4 and CD8. 1-way ANOVA and Tukey's multiple comparison test (between each experimental group and the matching control): (***) P<0.001.

The RAG2^−/−^ mice that we utilized for the above analysis are deficient in both T and B cells. We thus sought to further dissect whether the effects on the peritumoral fibrotic capsule could be attributed to only one of these lymphocyte populations. To achieve this, we depleted the B cells from a cohort of C57BL/6 mice by administering a mouse anti-mouse CD20 antibody, using vehicle (saline) treatment as control. In parallel, we treated another cohort of C57BL/6 with an armenian hamster anti-mouse CD3e antibody (or isotype control), in order to deplete T cells. Four days later, all mice received subcutaneously PANC02 tumor cells, whilst their T and B cell levels were regularly monitored (Figure 7). 3 weeks after tumor injection, we sacrificed all recipients and analyzed the formation of peri-tumoral capsules (Figure 7A, 7B). B cell depletion appeared to slightly reduce capsule formation, yet no significant effect was observed (Figure 7B, left segment). On the other hand, T cell depletion did lead to a significant reduction in collagen deposition in the peritumoral capsule (Figure 7B, right segment). This finding lends further support to our interpretation above, that T cells significantly contribute to the formation of a peritumoral fibrotic capsule. Nonetheless, it would not be prudent to exclude that B cells could also contribute to capsule formation; yet the weak effect observed will require far more extensive studies in order to provide a more definitive answer.

## DISCUSSION

Efficient adoptive cell therapy to combat established tumors depends upon a sufficient number of cytotoxic T cells, with high avidity for tumor antigen, reaching the tumor site without being dispersed in healthy tissue [14, 37]. The results presented here demonstrate that a substantial benefit can be obtained by ectopically expressing on the transferred T cells a chemokine receptor cognate for a chemokine that is strongly and differentially produced at the tumor site. Our approach significantly improves directed homing to the tumor, as demonstrated in two different experimental systems: in spontaneous lymph node metastases of prostate adenocarcinoma mice as well as in implanted E.G7-OVA lymphoma. As a result, a significant delay in tumor growth was obtained. Our proof-of-principle approach is novel in being applied to a spontaneous tumor model. By tailoring the receptor modification to match any chemokine identified in the metastasis, whichever this may be, the strategy could theoretically be applied to any unknown tumor. Indeed, it would be exceptionally suited to the treatment of diffuse metastasis, where a biopsy of a single accessible metastatic site could be used to identify the chemokine secretion signature of less accessible sites. This is highly relevant to prostate cancer, where diffuse metastatic relapse often occurs after the primary tumor has been surgically removed [38]. Unlike experimental ACT, which can be easily enhanced by the combination of several auxiliary boosting protocols, in the clinic ACT is often sub-optimal [1]. In such conditions, incremental improvements that can be added with minor protocol modifications, such as the addition of a second viral construct during the transduction of the specificity-conferring TCR, could be essential in enhancing the overall efficacy of the therapy.

We also found that the presence of prostate cancer metastasis in a lymph node was associated with increased collagen deposition. Whilst such a correlation is well-described in “fibrosis-prone” tumors [2, 8, 9], our finding led us to speculate that tumor fibrosis might be a phenomenon inherently linked to tumor presence *per se* rather than to specific “fibrosis-prone” tissues. Fibrosis is a physiological response that occurs during wound healing; intriguingly, the formation of tumor stroma is thought to share many characteristics with healing wounds, albeit with a chronic, non-resolving nature [33]. Fibrosis in the context of wound-healing is linked to persistent type-2 immunity [39] and appears to be dependent on T cell presence [34]. Type-2 (“alternative”) immunity during infections requires the presence of Th2 cells to maintain its chronicity [40]. In the liver, Th2 cells assist fibroblasts in promoting fibrosis [41]. In the context of tumors, where fibroblasts may be tumor-promoting FAP^+^ Cancer-Associated Fibroblasts [42], Th2 cells contribute to pro-tumoral inflammation in pancreatic [35] and breast cancers [43]. Whilst there is ample evidence that the collagen deposition *per se* is likely to be mediated by the combined action of tumor-infiltrating macrophages and cancer-associated fibroblasts [2, 44–47], the reports discussed above, taken together, hint that T cells (with a Th2 polarization) may play a role in the formation of pro-tumoral fibrosis. One can speculate that this may be occurring via their effect on the chronicity of the pro-fibrotic innate inflammatory response [40].

The experiments we present here give further support to the above conclusions, by showing that, in an ectopically injected pancreatic (thus fibrosis-prone) tumor model, T cell deficiency significantly reduces the extent of fibrosis and the ability of T cells to infiltrate the tumor. Our functional demonstration of impaired T cell infiltration in T cell-sufficient recipients contains the important caveat that we did not use externally administered labeled T cells in T cell-sufficient recipients as an additional control. Yet, as the only parameter measured was the gradient of the infiltrating cell distribution, which reflects the accessibility of the tumor, it is unlikely that this would result in a different distribution gradient, as indeed already existing T cells were impeded in their infiltration. Similarly, as the RAG2^−/−^ mice we utilized are T and B cell-deficient, we cannot formally exclude that B cells have a role in promoting peritumoral fibrosis. This would be consistent with their ability to induce production of pro-fibrotic cytokines [48, 49]. Nonetheless, in our antibody-mediated depletion experiments, removal of T cells had a stronger and significant effect, compared to removal of B cells. Thus fibrosis is, at least partially, dependent on T cell presence. To our knowledge, the T cell-dependent fibrosis formation that we observed has never been reported before. Partly, this could be attributed to the fact that many clinically-relevant ACT studies are performed in xenotransplantation-permissive immunodeficient models, which have no host T cells. Further, the duration of the treatment may be too short to allow development of pro-fibrotic Th2 cells.

Ever since the demonstration of T cell-mediated immunosurveillance [36], pro-inflammatory T cells have been considered an essential weapon in the battle against tumors. Indeed the central principle underlying ACT is their ability to attack the tumor mass or metastasis. For this, it is envisaged that they should be administered to patients in as “stem cell-like” a state as possible [1, 50], to guarantee their long-lasting *in vivo* functionality. As CD4^+^ T helper cells have been shown to significantly contribute to boosting anti-tumor cytoxicity [51], a co-transfer of CD4^+^ and CD8^+^ T cells can be envisaged as being part of optimized ACT protocols. Yet our results enable the speculation that a proportion of long-surviving CD4^+^ T cells transferred into a cancer patient could, theoretically and eventually, become polarized to a Th2 phenotype. These cells would thus assist in the formation of tumor-promoting fibrotic capsules, protecting any surviving tumor/metastatic foci, the very same targets that ACT is designed to combat.

The potential conflicting function of immune cells in their interaction with cancer is not a new concept, especially in innate immunity [52]. Yet T cell-mediated therapy studies occasionally neglect that pro-inflammatory T cells can be pro-tumoral [35, 43]. Our data demonstrates that tailored chemokine receptor modification can be used to enhance the homing efficacy of the transferred T cells in ACT. Yet our data also offers a hint that T cells present in the same organism are partly responsible for the fibrotic structures that may, in some cases, be protecting tumors from T cell attack. It could, therefore, be sensible to apply anti-fibrotic or anti-type 2 therapies [53] in combination with T cell-mediated ACT, so as to avoid that (some of) the cells that are being used therapeutically could end up contributing to the disease.

## MATERIALS AND METHODS

### Mice and tumor cell lines

TRAMP and C57BL/6J mice were sourced from Charles River Laboratories. Heterozygous TRAMP used in the experiments were obtained and screened as in [17]. Mice were treated in accordance with European legislation and local regulations. E.G7-OVA (ATCC CRL-2113) cells derived from murine lymphoma EL4 that express OVA constitutively (kindly provided by Dr. R. Bonecchi, Humanitas Clinical and Research Center, Italy) were cultured in RPMI 1640 medium with 2 mM L-glutamine, 1.5 g/L sodium bicarbonate, 4.5 g/L glucose, 10 mM HEPES, 1.0 mM sodium pyruvate, 0.05 mM 2-mercaptoethanol, 0.4 mg/ml G418 and 10% fetal bovine serum. HEK-293 cells derived from human embryonic kidney cells were cultured in the presence of high-glucose medium Iscove's Modified Dulbecco's Medium (IMDM) with L-glutamine, in the presence of 0.001% 2-mercaptoethanol, 25 U/ml penicillin and streptomycin and 10% fetal calf serum. PANC02 cells derived from murine pancreas ductal adenocarcinoma [54] were cultured in RPMI 1640 medium with 2 mM L-glutamine, 1.5 g/L sodium bicarbonate, 4.5 g/L glucose, 10 mM HEPES, 1.0 mM sodium pyruvate, 0.05 mM 2-mercaptoethanol and 10% fetal bovine serum.

### CD8^+^ T isolation and activation

CD8^+^ T cells were isolated from spleens and inguinal lymph nodes of C57BL/6 mice using CD8a^+^ T cell Isolation Kit II (Miltenyi Biotec). The purity obtained was assessed by staining for anti-mouse CD8-PE (eBioscience) and analyzing on FACS (BD) and was always higher than 90%. CD8^+^ T cells were plated overnight at 1.5 × 10^6^ cells/well in IMDM containing 10 ng/ml rmIL-2, 10 ng /ml rmIL-7, 10 ng/ml rhIL-15 (all PeproTech) in 24 well plates previously coated with 2 μg/ml NA/LE anti-mouse CD3ε (BD) and 1 μg/ml NA/LE anti-mouse CD28 (BD Pharmingen).

### Retroviral transduction of T cells

The day before transfection, 2.4×10^6^ HEK-293 cells were plated onto 6 well plates (BD) using 12 ml of Iscove's Modified Dulbecco's Medium (IMDM) high glucose with L-glutamine, 0.001% 2-mercaptoethanol and 10% fetal calf serum. Transfection of retroviral constructs was performed in Optimem (Gibco) with Lipofectamine 2000 at 1.6% (Invitrogen), using 2 μg of packaging vector pCL-Eco (Imgenex) and combination of the following plasmids: i) m6p-egfp[Blast] [11] Green Fluorescent Protein (EGFP) control, (kind gift of Dr Randow and Dr Betz, MRC-LMB, Cambridge, UK) ii) m5p-CCR2-2A-egfp, created by inserting a T2A self-cleaving sequence in between a mouse CCR2 (cloned from cDNA) and EGFP [55] iii) pMX-SV40α-IRES-SV40β, encoding a TCR specific for SV40 Large T antigen [27] (kind gift of Dr Schumancher NKI, Amsterdam, NL) and iv) TCR OTI-2A.pMIG II, encoding a TCR specific for ovalbumin [56] (Addgene). 48 hours post-transfection, filtered viral supernatants supplemented with fresh IL-2/IL-7/IL-15 were added to T cells (at 1.5 × 10^6^ cells/ml) that had been purified and activated the previous day. The cellular suspension was seeded into Retronectin (Takara Bio) coated plate (2ml/well), spun at 1200g for 1h at 30°C and incubated overnight at 37°C/5% CO2. The following day T cells were collected, washed and allowed to rest in medium containing 10% FCS and IL-2/IL-7/IL-15 in 24 well plates for 48 hours at 37°C/5% CO2. HEK-293 transfection and CD8^+^ T cell transduction efficiency were assessed on FACS by examining EGFP expression as well as by staining for the T cell receptor (TCR) constructs used for ACT: for the SV40 Large T antigen-specific TCR, anti-Vbeta9 TCR (eBioscience) was used; for the Ovalbumin-specific TCR coexpression of anti-Valpha2 TCR and anti-Vbeta 5.1/5.2 TCR (eBioscience) was assessed. The cells were then used for *in vitro* or *in vivo* assays.

### Immunohistochemistry for SV40 TAg detection

Sample sections on slides were deparaffinized and hydrated through a descending scale of alcohols. Antigen retrieval was performed using DIVA (Biocare Medical). Sections were cooled, washed with PBS (Lonza) containing 0.05% Tween 20 (Sigma). Endogenous peroxidase was blocked by incubation with Peroxidase I (Biocare Medical) for 20 min at room temperature (RT) and nonspecific sites were blocked with Rodent Block (Biocare Medical) 20 min at RT. The sections were incubated for 1h at RT with rabbit anti SV-40 (Santa Cruz Biotech V-300) diluted 1:250, washed, incubated for 30 min at RT with anti-rabbit HRP polymer (Biocare Medical). Sections were incubated with DAB (Biocare Medical), counterstained with hematoxylin, dehydrated through an ascending scale of alcohols and xylene, and mounted with coverslips using Eukitt (Fluka).

### Analysis of *in vivo* homing

Recipient TRAMP mice received cells i.v. that were either transduced with i) pMX-SV40α-IRES-SV40β and m6p-egfp[Blast] or ii) with pMX-SV40α-IRES-SV40β and m5p-CCR2-2A-egfp. The same number of EGFP^+^ cells (control or CCR2-expressing) were injected in all recipients (5×10^5^ to 10^6^, varying between but not within experiments). After 24 hours, inguinal and para-aortic lymph nodes and spleen were harvested to assess homing of the transferred EGFP^+^ cells within these tissues. Single cell suspensions of spleens were obtained using 70μm cell strainers. Inguinal and para-aortic lymph nodes were dissected and incubated in 0.005% collagenase IV (Sigma-Aldrich) at 37°C for 1h and in IMDM medium with 10% FCS for a further 2 hours at 37°C/5% CO2. Cell suspensions were assessed by flow cytometry for EGFP expression, after exclusion of dead cells by Live/Dead fixable Aqua kit (Life Technologies). An aliquot of the cells was kept for real-time qPCR analysis of SV40 Large T Antigen expression, to identify whether the lymph node was indeed metastatic.

To evaluate EGFP^+^ cell infiltration within E.G7-OVA tumors, tumor sections were incubated for 1h at RT with rabbit anti-EGFP (A10263, Invitrogen) and for 1h at RT with Streptavidin-peroxidase (Biocare Medical). Immunoreactivity was visualized with 3,3-diaminobenzidine (DAB, Biocare Medical). All sections were counterstained with hematoxylin and photographed with an Olympus BX53 microscope with a digital camera. EGFP^+^ cells were determined with ImageJ software and expressed as number of positive cells divided by the total section area.

For co-staining of T cells and tumor cells, the TRAMP SV40 TAg^+^ lymph node sections were incubated for 1h at RT with rabbit anti-EGFP (A10263, Invitrogen) and rabbit anti-SV40 T (sc-20800, Santa Cruz Biotechnology) and then for 15′ at RT with Streptavidin-peroxidase (Biocare Medical). Endogenous peroxidase was blocked by incubation with Peroxidased I (Biocare Medical) for 20 min at RT and nonspecific sites were blocked with BSA (Euroclone) 1h RT. Immunoreactivity for anti-EGFP was visualized with DAB. After wash with TBS, the sections were incubated for 30′ with MACH 4 Universal AP Polymer Kit. Endogenous phosphatase was blocked by incubation with Levamisole (Dako) for 5′RT. Immunoreactivity for anti-SV40 T was visualized with Ferangi Blue (Biocare Medical).

### *Ex vivo* 2-photon microscopy of lymph nodes

Lymph nodes were harvested and fixed in PFA 4% overnight. Images were captured with a TrimScope II multiphoton microscope (LaVision) using a 20x/1.0 Olympus LUMPLFL-WI objective. Excitation was provided by a Chameleon Ultra II Ti:Sa laser tuned at 840nm for collagen second harmonic signal (SHG) or at 920nm for EGFP excitation. Three-dimensional mosaic stacks of the first 20-50 μm were captured with 5-10% overlap and 2 μm z-step. Raw images were saved as OME TIFFs, then reconstructed with ImageJ/FIji Stitching Plugin [57]. Images are displayed as a maximum-intensity projection of 10-μm-z-stack. Collagen density analysis was conducted on ImageJ/Fiji. SHG channel was thresholded with fixed threshold levels, followed by image conversion to binary. For each slice, the capsule region was selected with hand drawing tool to exclude background and artifact regions. Drawing error was taken into account with a 2 pixel error on the hand-drawn perimeter. White/black pixel density was measured on these regions. Cells in each lymph node were counted with Imaris software (Oxford Instruments) and Spot Detection module. To calculate background threshold, we measured average background autofluorescence in wild-type lymph nodes from untreated mice with ImageJ. Lymph node volume was calculated with Cavalieri Principle approximation, multiplying each area by the z-step.

### Chemokine expression by Real-Time PCR

Tumor masses and prostates were harvested in RNA Later (Ambion) and homogenized with Tissue Lyser II (QIAGEN). Total RNA was extracted with RNeasy Mini Kit (QIAGEN). Inguinal lymph nodes and paraortic lymph nodes were digested with Collagenase IV (Sigma-Aldrich) and total RNA was extracted from 1×10^5^ cells using RNeasy Micro Plus Kit (QIAGEN). After quantification, RNA was reverse-transcribed into cDNA using High Capacity cDNA Reverse Transcription kit (Applied Biosystems). Within each experiment the same amount of cDNA was used, in the range 14-60 ng/reaction. Real-time qPCR was performed using TaqMan gene expression assays (CCL2 ID: Mm00441242_m1, CCL5 ID: Mm01302427_m1, CXCL12 ID: Mm00445553_m1, CXCL9 ID: Mm00434946_m1, CXCL10 ID: Mm00445235_m1, CXCL11 ID: Mm00444662_m1 and Rn18S ID: Mm03928990_g1). SV40 Large T antigen expression was assayed using a custom-made commercial TaqMan gene expression assay.

### T cell-mediated cytotoxicity assay

Cell-mediated cytotoxicity was assayed as in [58], using CD8^+^ T cells from C57BL/6 spleens and inguinal lymph nodes, transduced with TCR OTI-2A.pMIG II/m6p-egfp[Blast] and TCR OTI-2A.pMIG II /m5p-CCR2-2A-egfp as effector cells and the E.G7 cell line as target cells. Target cells were labeled with CellTracker™ Orange CMTMR Dye (Life Technologies) and cell death was assessed by 7-aminoactinomycin D (7-AAD) (Life Technologies) on FACS (BD).

### Costimulation and migration assay

CD8^+^ T cells were treated according to the preparative protocol for ACT, as described above. Following the resting period in IL-2/IL-7/IL-15, they were plated at 2 × 10^5^ cells/well in flat 96-well plates and were either unstimulated or treated with CCL2 (100ng/mL) in fluid phase and/or pre-coated anti-CD3e and/or anti-CD28, using clones and concentrations as above. The cells were harvested for analysis after a further 18 hours and stained using anti-CD69 (H1.2F3; BD) and anti-CD25 (BPC61.5; eBiosciences). For the migration assay, Costar (Corning) 24-well plates with 3 μm pore size were used. Transduced T cells were collected, counted and resuspended in IMDM BSA 0,1% at 3 × 10^6^ cells/mL. 100 μL of cells were placed in the upper chamber. The bottom chamber was filled with 500 μL of IMDM BSA 0,1% with or without CCL2 (100 ng/mL). Cells were incubated at 37°C for 2 h to allow migration. Migrated cells were collected from the bottom chambers and counted using FACS (BD).

### *In vivo* tumor growth evaluation

C57BL/6 mice were injected on the right flank with 5×10^5^ E.G7-OVA cells. The tumors were allowed to grow for 24 days. Mice were treated in one of three groups: a) not treated b) treated with CD8^+^ T cells transduced with TCR OTI-2A.pMIG II and m6p-egfp[Blast] c) treated with CD8^+^ transduced with TCR OTI-2A.pMIG II and m5p-CCR2-2A-egfp. The same number of TCR OTI-2A.pMIG II expressing T cells were injected for both groups. 7×10^5^ transduced CD8^+^ T cells per mouse were administered intravenously on day 8 and day 16 after tumor challenge. On day 16 the tumor masses were monitored by radiance emission of specific *in vivo* tumor marker IntegriSense 750 (Perkin-Elmer) [59], injected the previous day. The radiance was measured by IVIS Lumina (Perkin-Elmer). On day 24 the tumor masses were monitored again immediately after sacrifice and harvesting. Grubb's test was applied on the entire data set, identifying two spurious outliers.

### Assessment of collagen content in PANC02 tumors

C57BL/6 or RAG2^−/−^ mice were injected subcutaneously on the right flank with 5×10^5^ PANC02 cells. The tumors were allowed to grow for 3 weeks. Harvested mouse tumors were fixed in 4% formalin, processed for paraffin embedding and sectioned at 3μm. Tissue collagen content was determined by staining sections in Masson trichrome (VWR International). Images of Masson's trichrome staining were acquired with the Olympus VS120 dotSlide and analyzed to quantify the fibrotic areas, measured as collagen-stained area as a percentage of total section area, within each region of interest. The slides were additionally analyzed using SHG 2-photon microscopy, as described above.

### T cell density distribution analysis

Tumors from C57BL/6 or RAG^−/−^ mice that had received adoptively transferred Cell Tracker Orange CMTMR (Life Technologies)-labeled CD3^+^ cells were fixated in 4% PFA, processed for paraffin embedding and sectioned at 3μm. Samples were then either DAB stained for CD3 and acquired with an Olympus VS120 dotSlide slide scanner or acquired as is at 2-photon microscope for CMTMR fluorescence and SHG. Whole tumor slides were acquired and reconstructed with 20x magnification as previously described. Images were analyzed with a custom ImageJ plugin to generate a distribution of cells as a function of distance from tumor margin. Cells recognized by the algorithm were thresholded by signal intensity and with an upper size limit of 200 μm^2^.

For the analysis of the contribution of T cells versus B cells to peritumoral fibrosis, C57BL/6 mice were injected intra-peritoneally with 250 μg B cell-depleting mouse anti-mouse CD20 antibody (clone 5D2, Genentech Inc.) or vehicle (PBS). Alternatively, they were injected with 250 μg T cell-depleting armenian hamster anti-mouse CD3e (BioXCell, InVivo plus, clone 145-2C1) or isotype control (BioXCell, InVivo Plus Armenian Hamster IgG). 4 days later (“day 0”), they received 7.5×10^5^ PANC02 cells per mouse subcutaneously. T cell and B cell levels were monitored via FACS for CD19 (1D3, eBioscience), CD4 (RM4-5, BD) and CD8 (53-6.7, BD) on day −5, −1, 16 and 21. On day 21, harvested tumors were analyzed by staining sections with Masson's trichrome, as above.

### Statistical analysis

Statistical analysis was performed with GraphPad Prism software, using unpaired t-test/1-way ANOVA for Gaussian or Mann Whitney/non-parametric ANOVA tests for non-Gaussian distributions, after normality testing. Grubb's test was applied to identify spurious outliers.

## SUPPLEMENTARY MATERIAL


