# Supplementary material for: Tailored chemokine receptor modification improves homing of adoptive therapy T cells in a spontaneous tumor model
Source: Oncotarget. 2016 May 10;7(28):43010–26. doi: 10.18632/oncotarget.9280 (PMC5190004; doi:10.18632/oncotarget.9280)
Supplement: Supplementary file 1 [file oncotarget-07-43010-s001.pdf]

Tailored chemokine receptor modification improves homing of adoptive therapy T cells in a spontaneous tumor model

Supplementary Material

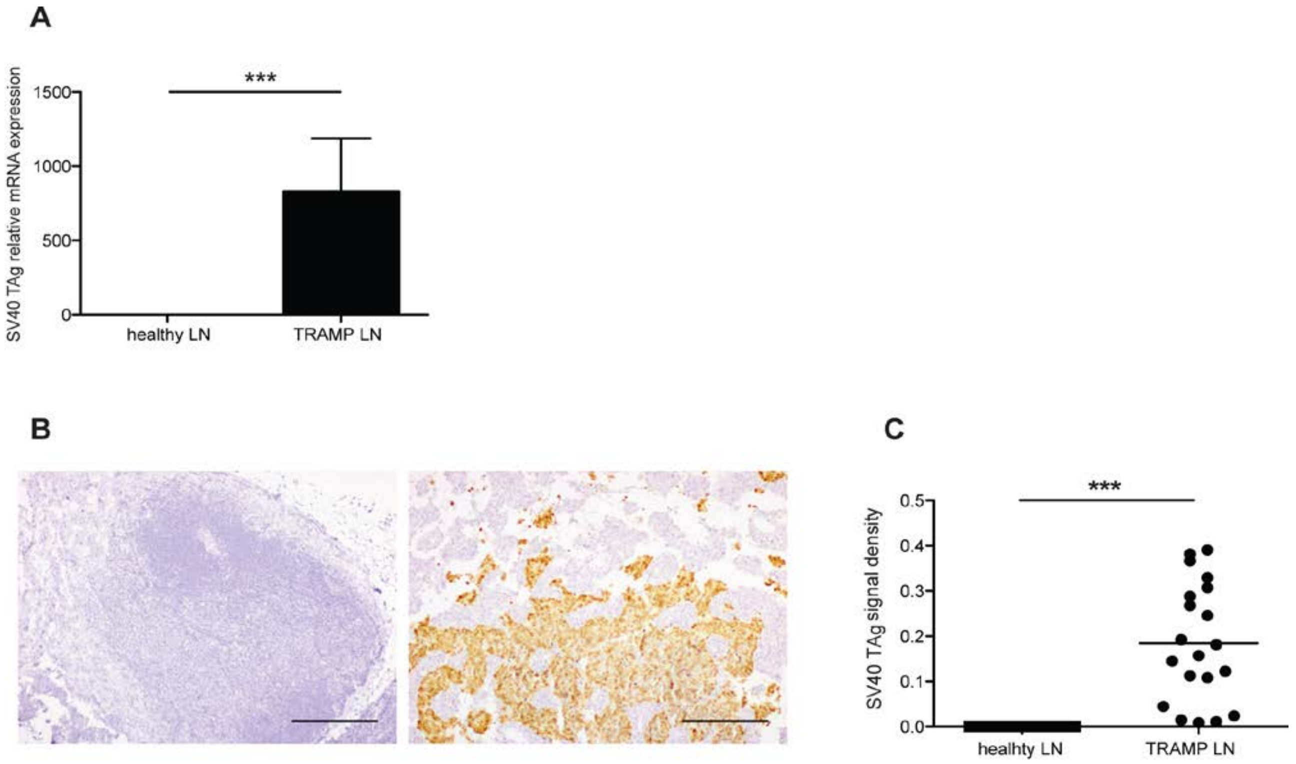

Supplemental Figure 1

A: Representative mRNA expression of SV40 TAg genetic marker of TRAMP tumors, via real-time qPCR. Fisher’s test (zero signal versus positive signal): (***) P<0.001. **B:** Representative images of immunohistochemical analysis of SV40 TAg on mouse lymph nodes from C57BL/6 (ctrl) or TRAMP mice bearing lymph node metastasis: Brown staining SV-40^{+} cells. Original Magnification 20x. Scale bars: 100 μm.

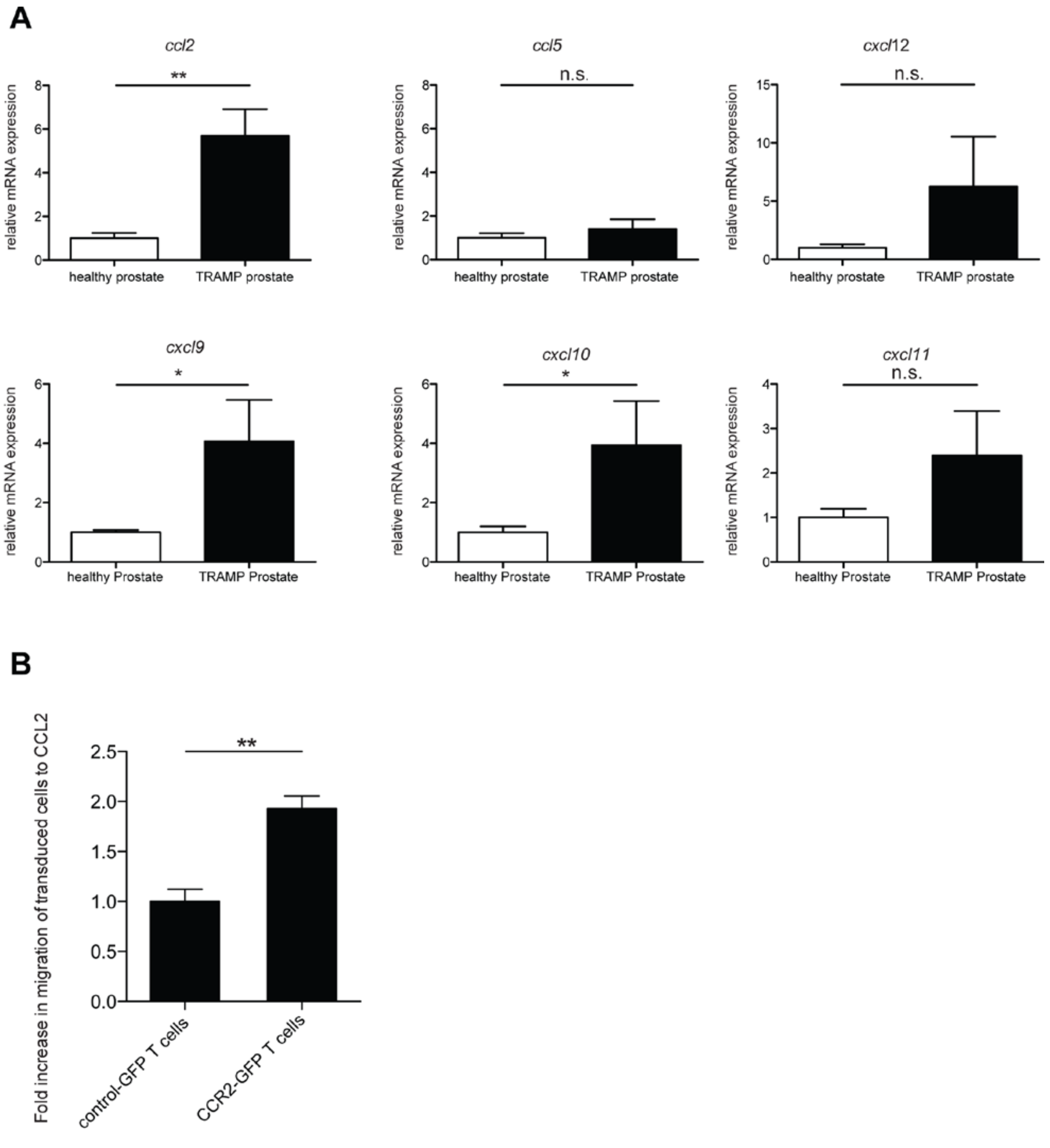

Supplemental Figure 2

A: Chemokine expression in TRAMP primary tumors. *Ccl2, ccl5, cxcl12, cxcl9, cxcl10 and cxcl11* mRNA relative expression in TRAMP or healthy prostate; Mann Whitney test: (*) $P<0.05$; (**) $P<0.01$; (ns) $P>0.05$. **B**: Transduction with CCR2 construct improves *in vitro* migration to CCL2. CCR2-transduced or control-transduced T cells were allowed to migrate toward CCL2 in a transwell assay. The migrated T cells were analyzed by FACS and the fold increase in migration is shown. Unpaired t test: (**) $P<0.01$.

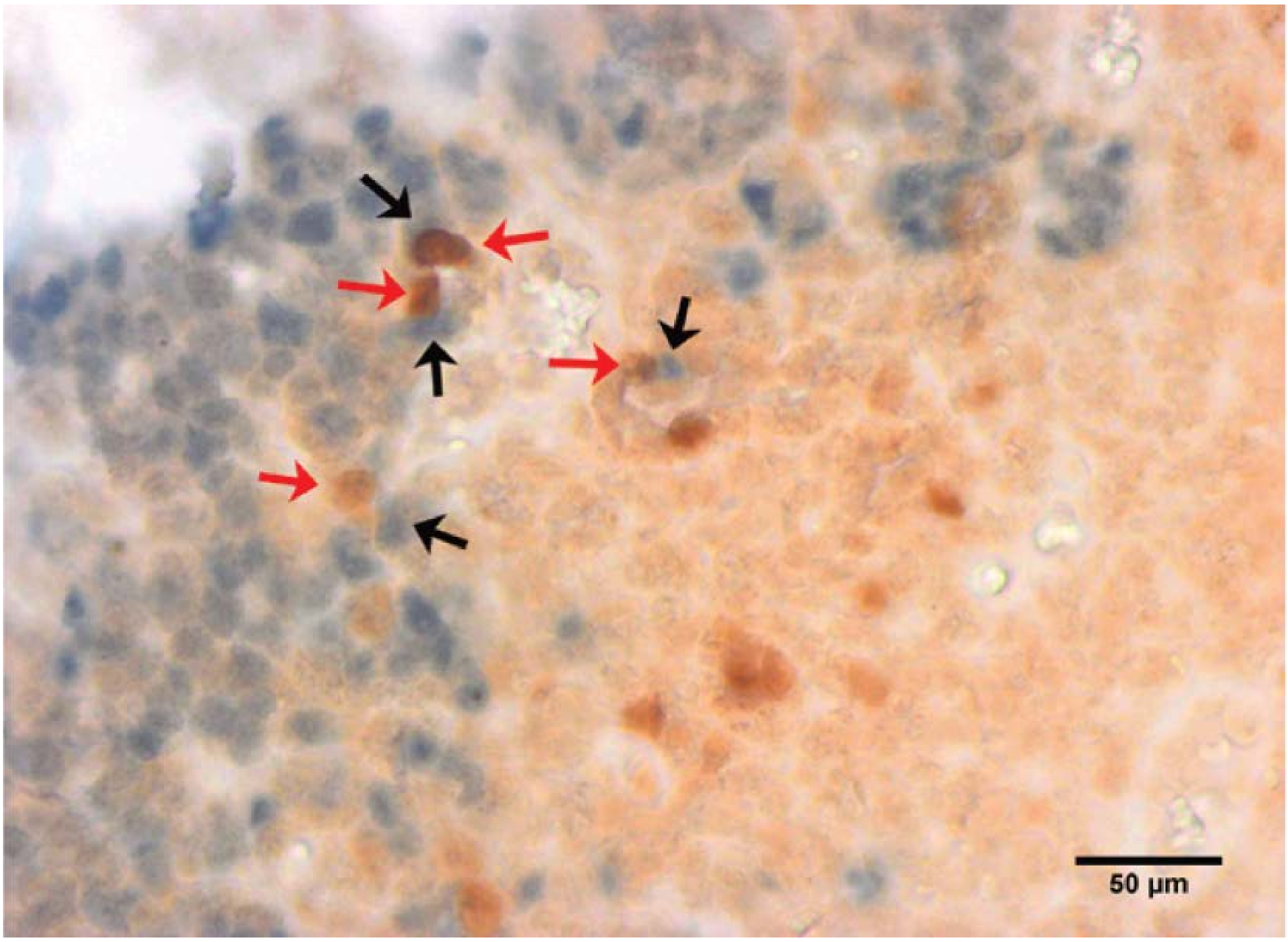

Supplemental Figure 3

CCR2-trasduced $CD8^+$ T cells in the metastatic lymph node are in contact with tumor cells. Representative image of immunohistochemical analysis of TRAMP SV40 TAg^+ metastatic lymph node sections from a recipient of $CD8^+$ T cells transduced with GFP-CCR2 and a TCR specific for SV40 TAg. Brown staining/red arrows: GFP^+ cells (GFP-$CCR2^+$ $CD8^+$ T cells). Blue staining/black arrows: SV40 TAg^+ (tumor) cells.

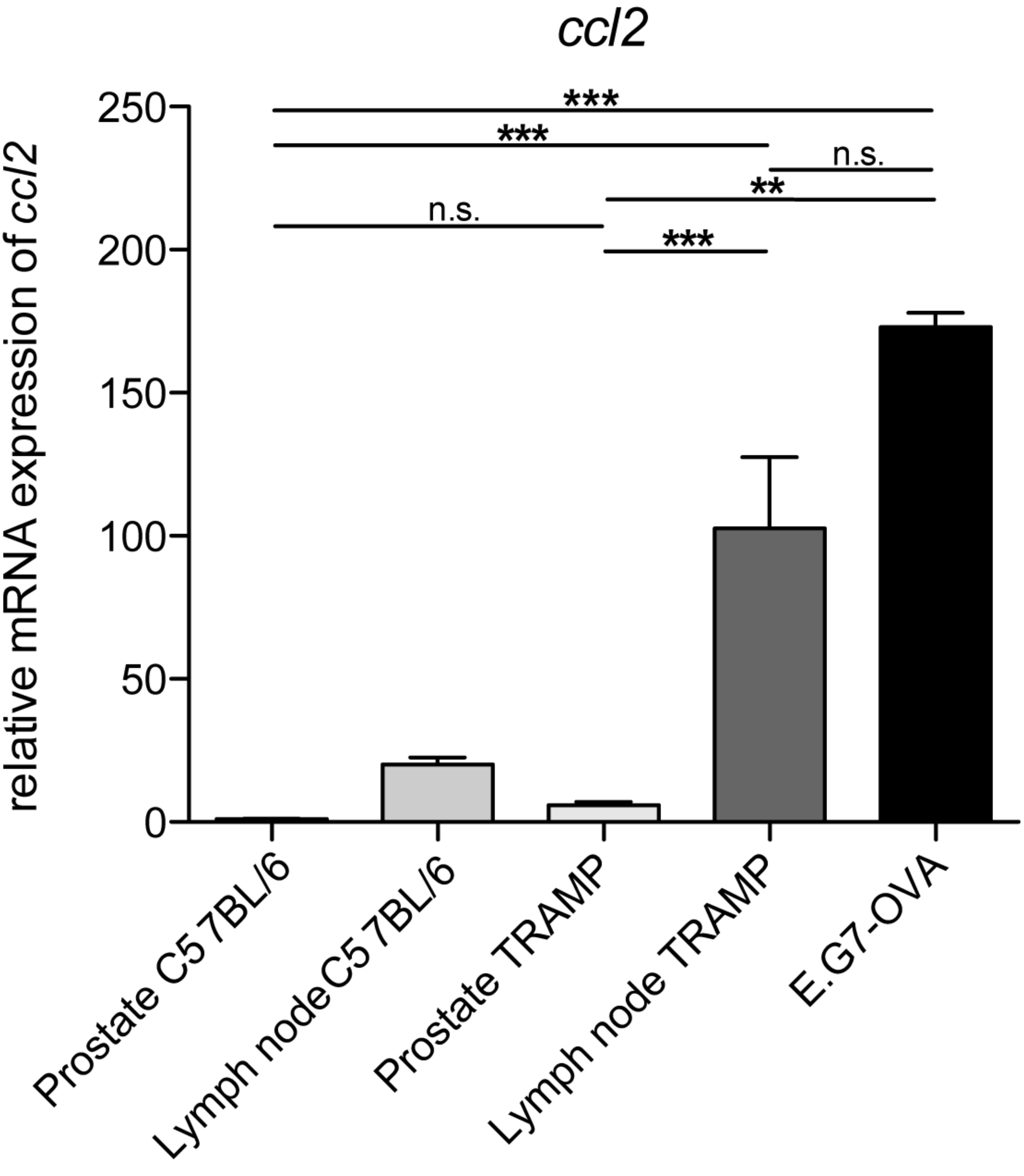

Supplemental Figure 4

Ccl2 mRNA expression via qPCR on healthy C57BL/6 prostate tissue, healthy C57BL/6 lymph nodes, TRAMP prostate tumor and lymph nodes, E.G7-OVA lymphoma-derived implanted tumors. Data shown normalized to healthy prostate tissue; Non parametric 1-way ANOVA and Dunn's multiple comparison test: (***) P<0.001; (**) P<0.01; (ns) P>0.05.

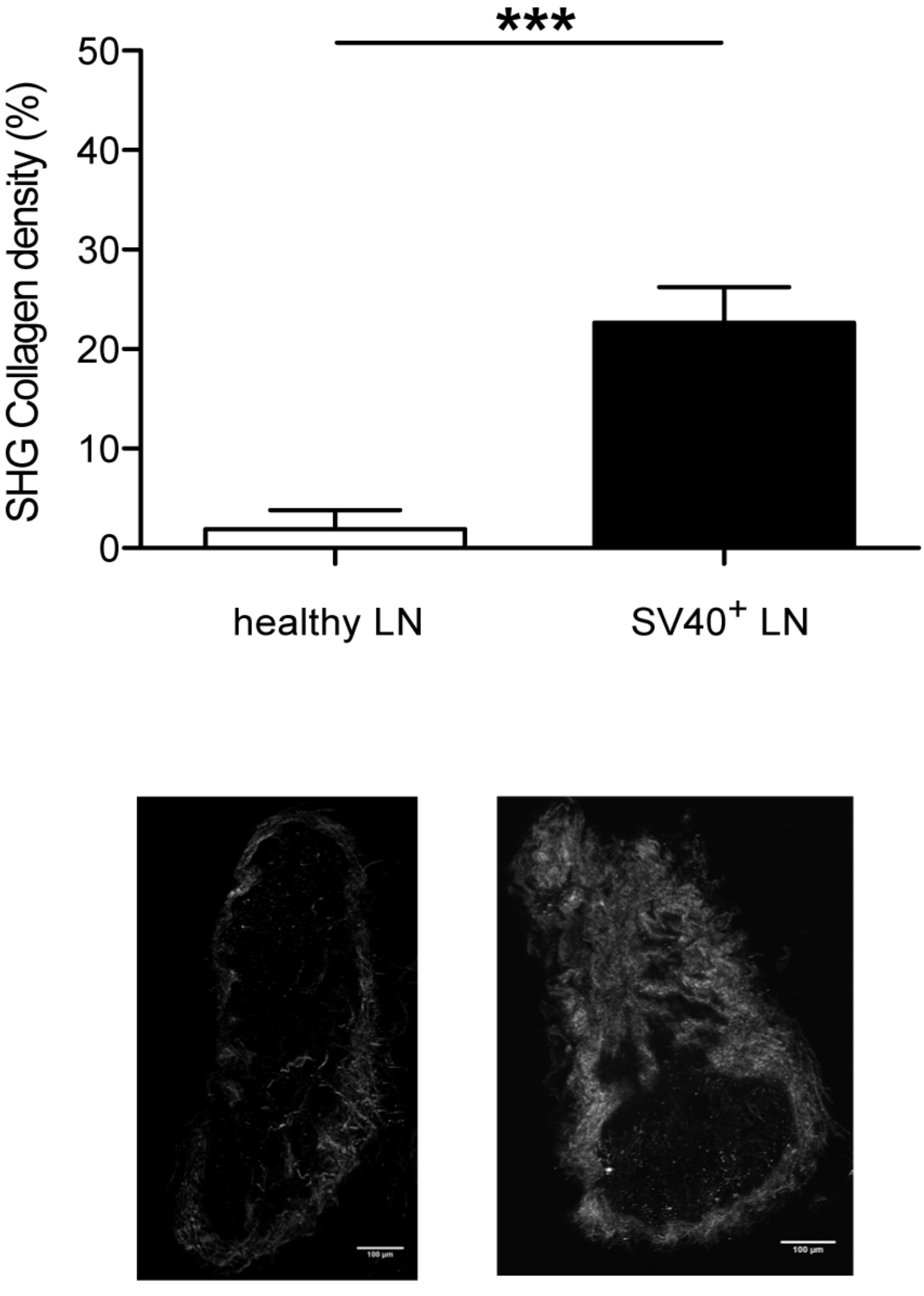

Supplemental Figure 5

Metastatic lymph nodes show higher collagen density compared to healthy lymph nodes. Collagen density was evaluated by second harmonic generation (SHG) in 2-photon microscopy. Graph of collagen density in SV40 TAg^{+} lymph nodes (n=9) or healthy lymph nodes (n=12); unpaired t test: (***) P<0.001 (top). Representative images of SHG (shown in white) in SV40 TAg^{+} and healthy lymph nodes (bottom).

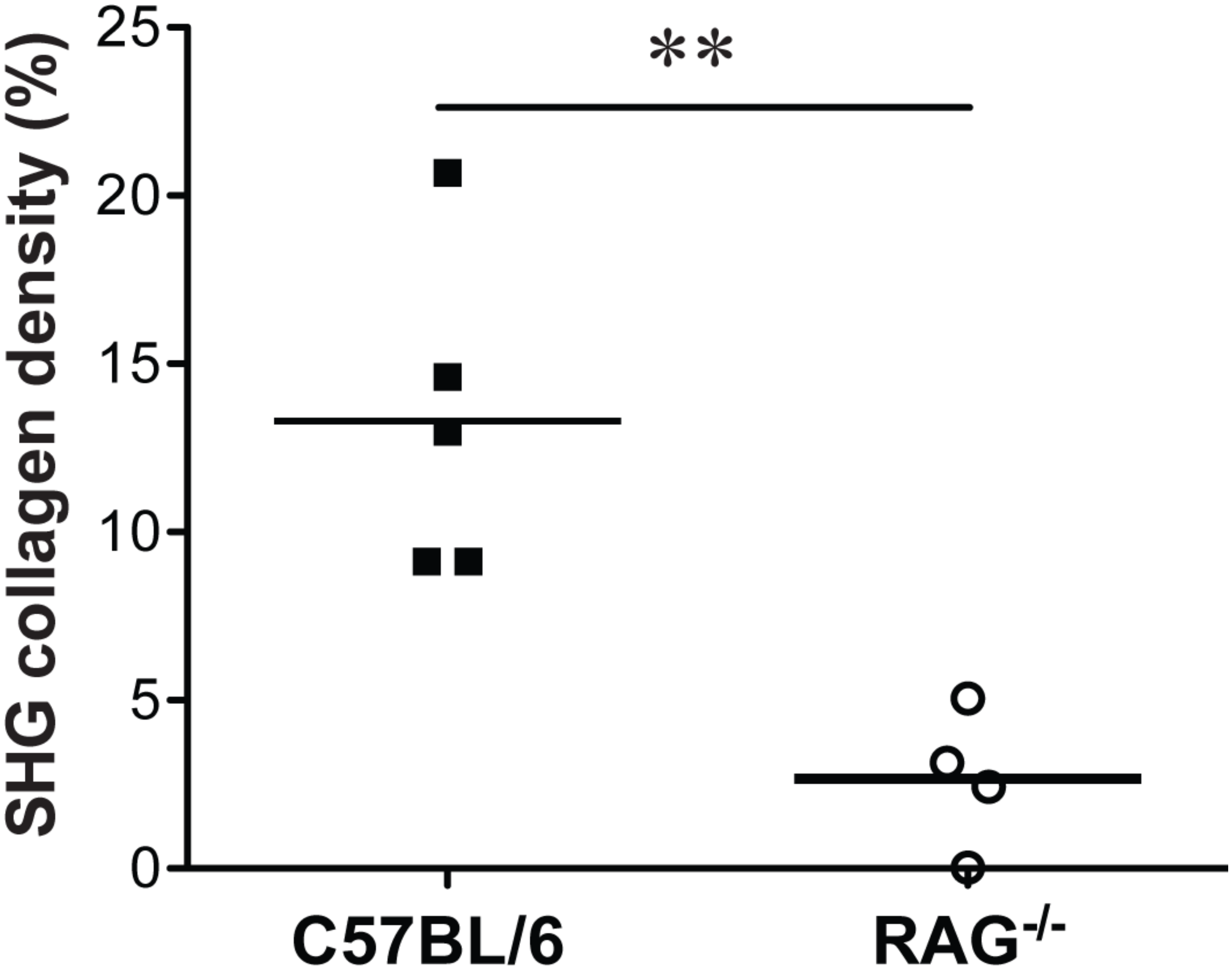

Supplemental Figure 6

Host T cell presence favors fibrosis formation. Collagen density in the presence (C57BL/6 recipients) or absence (RAG$^{-/-}$ recipients) of host T cells was evaluated by second harmonic generation (SHG) in 2-photon microscopy; unpaired t test with Welch's correction: (**) P<0.01; non-parametric t test (Mann-Whitney test; on the same data set) (*) P<0.05.

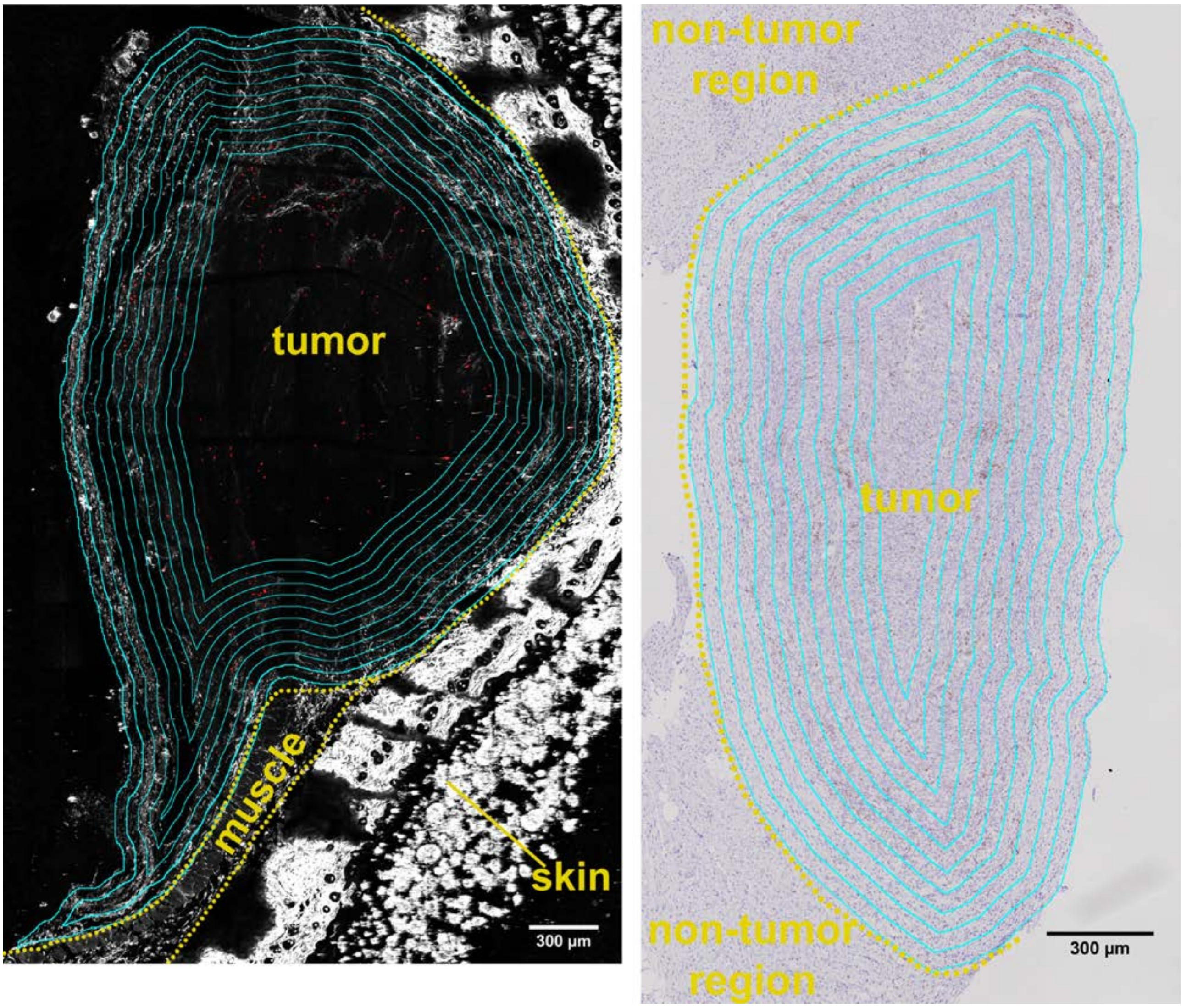

Supplemental Figure 7

Representative images of concentric regions of analysis of T cell distribution across the capsule. The image on the left is a 2-photon image of a tumor grown in a RAG$^{-/-}$ recipient that received CMTMR-labeled T cells 3 days prior to harvesting. Red stain indicates T cell presence, white stain indicates collagen presence via SHG. The concentric regions start at the outer boundary of the peri-tumoral capsule and span 450 µm into the tumor. The image on the right is an immunohistochemical analysis of a tumor grown in C57BL/6 recipients, which are T cell-sufficient. Brown staining indicates T cell presence. The concentric regions start at the outer boundary of the peri-tumoral capsule and span 450 µm into the tumor.
